# Interleukin 15 Pharmacokinetics and Consumption by a Dynamic Cytokine Sink

**DOI:** 10.3389/fimmu.2020.01813

**Published:** 2020-08-13

**Authors:** John A. Hangasky, Thomas A. Waldmann, Daniel V. Santi

**Affiliations:** ^1^ProLynx, San Francisco, CA, United States; ^2^Lymphoid Malignancies Branch, Center for Cancer Research, NCI, NIH, Bethesda, MD, United States

**Keywords:** cytokine, immuno-oncology, NK cells, CD8^+^ T cells, target-mediated drug disposition, interleukin-15, pharmacokinetics

## Abstract

Interleukin-15 (IL-15) is crucial for the proliferation and survival of NK and CD8^+^ T memory cells, and of significant interest in immuno-oncology. Immune cell expansion requires continuous IL-15 exposure above a threshold concentration for an extended period. However, the short t_1/2_ of IL-15 makes this impossible to achieve after a single injection without a high C_max_ and toxicities. The most effective way to deliver IL-15 is continuous intra-venous infusion, but this administration mode is impractical. Efforts have been devoted to developing IL-15 agonists which after a single injection maintain the cytokine in a narrow therapeutic window for a long period. Enigmatically, although the half-life extension technologies used often extend the half-life of a protein to 1 or more weeks, the modified IL-15 agonists studied usually have systemic elimination half-lives of only a few hours and rarely much longer than 1 day. These short half-lives—common to all circulating IL-15 agonists thus far reported—can be explained by a dynamic increase in clearance of the agonists that accompanies target immune cell proliferation. What is needed is an IL-15 agonist that is as effective as continuous intravenous infusion, but with the convenience and acceptance of single injections at 1-week or longer intervals.

## Introduction

**Figure 1:**
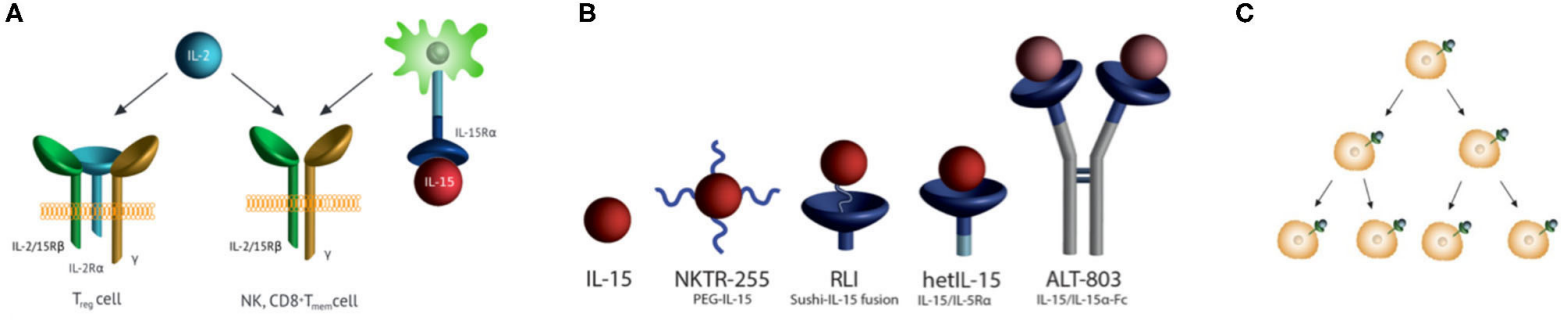
IL-15 receptor, receptor agonists, half-lives, and immune cell proliferation. **(A)** IL-15 and IL-2 receptor specificities. IL-15 is trans-presented in association with IL-15Rα to Rβ,γ subunits also used by IL-2. Trans-presentation of IL-15/IL-15Rα to responding cells leads to the activation of JAK1/3 which phosphorylate STAT3/5 to prompt transcription of IL-15-modulated genes. IL-2 binds to cells with the same Rβ,γ subunits, as well as the high-affinity trimeric IL-2 Rα,β,γ receptor on T_Reg_ cells. **(B)** IL-15R agonists in human clinical trials. (i) NKTR-255 is a PEGylated IL-15 (7), (ii) RLI is a fusion of the C-terminus of the IL-15Rα Sushi domain to the N-terminus of IL-15 via a 20 AA linker (8), (iii) HetIL-15 (aka NIZ985) is a heterodimeric complex of IL-15 and the extracellular Rα (9), and (iv) ALT-803 (aka N-803) has IL-15N72D bound to a Fc –IL-15Rα Sushi domain fusion (10, 11). **(C)** Receptor-mediated cell proliferation and increased cytokine consumption. The cytokine binds its extracellular receptor, signals to cause cell proliferation, and is endocytosed with variable degrees of destruction. With proliferating cells consuming more of the cytokine, clearance increases as target immune cells expand.

Interleukin 15 (IL-15) is a ~14 kDa four-α-helix protein belonging to a family of six interleukins that use a common cytokine-receptor γ-chain (1). The cytokine is crucial in the proliferation, maintenance, and survival of NK and CD8^+^ T memory cells, and is of major interest in immuno-oncology (2). IL-15 stimulates immune cell responses through the same dimeric IL-2/15Rβ,γ receptor complex as IL-2 (Figure 1A), but the two cytokines exhibit functionally distinct activities due to their private α-receptor subunits. As a consequence, either IL-15 or IL-2 stimulates NK and CD8^+^ T cells, but only IL-2 binds to and stimulates regulatory T cells that possess the IL-2Rα in a trimeric Rα,β,γ complex. IL-15 is expressed in association with its high affinity IL-15Rα on the surface of IL-15-producing cells and is trans-presented to immune cells that express dimeric IL-2/15Rβ,γ subunits (3). Once bound to target NK and memory CD8^+^ T cells, IL-15 stimulates their proliferation, and supports their survival (4, 5). Despite dramatic augmentation of NK cells and CD8^+^ T cells, IL-15 has minimal anticancer activity as a single agent. However, in combination with other immuno-oncology agents, it shows significant efficacy and it is in this setting that IL-15 will likely find success (6).

The pharmacokinetics and pharmacodynamics of IL-15 agonists are profoundly impacted by target-mediated drug disposition (TMDD) wherein the cytokine is consumed and cleared by target immune cells (12–14)—a “cytokine sink.” At high initial IL-15 concentrations, target cells are saturated with cytokine—(i.e., capacity-limited TMDD)—and clearance of the agonist is dominated by renal and/or metabolic elimination. IL-15 subsequently causes a time-dependent proliferation of target immune cells and commensurate time-dependent increase of cytokine consumption and clearance; once expanding target cells exceed the cytokine concentration and *K*_d_ of the IL-15–Rβ,γ the pharmacokinetics manifest as TMDD. The purposes of this review are to consolidate and scrutinize dispersed reports of the pharmacokinetics of IL-15 agonists, and to evaluate the evidence for generation of a dynamic cytokine sink that expands concomitantly with the proliferation of IL-15 target immune cells.

### Target Immune Cell Proliferation and Maintenance

NK and memory T cell proliferation requires continuous IL-15 exposure at a level above a threshold concentration for an extended period (4, 5). However, with a t_1/2_ of only ~2.5 h in the human it is impossible to achieve long exposure of IL-15 after a single IV injection without a very high C_max_ and resultant toxicities (15). For example, a bolus IV dose of 3 μg/kg IL-15 is necessary to maintain serum IL-15 at ≥1 pM – ~10-fold lower than the *K*_d_ of IL-15 for IL-2/15Rβ,γ – for a 24 h period but gives a C_max_ of ~3,000 pM, some 30-fold higher than the 100 pM C_max_ with a 0.3 μg/kg MTD (15). A possible and popular solution to this problem is to simply extend the t_1/2_ of the cytokine. This would concurrently flatten the *C* vs. *t* plot, enable prolonged exposure to optimize efficacy, and reduce the C_max_ peak responsible for toxicities.

Continuous IV (CIV) infusions of therapeutics are often safer than bolus injections since the flat *C* vs. *t* profile is absent the high C_max_ peak often associated with toxicity. Indeed, CIV infusion is the most efficient delivery method for IL-15, requiring low doses and resulting in large increases of target immune cells (16). In humans, 2 μg IL-15/kg/day of CIV infusion for 10 days led to a 5-fold increase in circulating CD8^+^ T cells, a 38-fold increase in the total NK cells, and a massive 358-fold increase in CD56^bright^ NK cells. Usually, infusing a drug at a constant rate that is balanced with its rate of elimination results in maintenance of a constant serum level. However, with CIV infusion of IL-15 serum levels decreased over the time of infusion—starting at ~360 pM and lowering to ~70 pM by the end of the infusion period—indicating a time dependent increase in IL-15 clearance. This is in accord with an expanding cytokine sink where the initial lower clearance rate reflects capacity-limited TMDD (12, 14) but which after target expansion to about 10^−9^ M presents a large receptacle exceeding both IL-15 concentration and the *K*_d_ of the IL-15-Rβ,γ complex; under saturating sink conditions, the clearance of IL-15 would be maximal. An interesting transition of the IL-15 dose-exposure relationship occurs between infusion of 0.5- and 1 μg/kg/day whereby a doubling in dose results in an >45-fold increase in serum IL-15. This further supports a conversion from capacity-limited TMDD to a clearance mechanism whereby the IL-15 exceeds the capacity of the cytokine sink. Notwithstanding, CIV infusion of IL-15 for 10 days is an inconvenient, expensive mode of long-term drug administration and is unacceptable to both patients and physicians. It would be desirable to have an IL-15 agonist that could be administered as single injections at intervals of 1 or more weeks.

### Pharmacokinetics of IL-15 Agonists

There have been extensive efforts to develop potent, long-acting IL-15 receptor agonists—referred to as “super-agonists” (17)—which after a single injection maintains the agonist in a narrow therapeutic window for a long period. Figure 1B depicts IL-15R agonists in clinical trials that have reported pharmacokinetic data, and Table 1 presents their reported t_1/2_ values in different species with different routes of administration. The approaches used to increase the t_1/2_ of IL-15 generally involve increasing the molecular size to reduce renal elimination; they include incorporation of part of the IL-15Rα and/or attachment to PEG or Fc to the cytokine. There are also current investigations—not covered here—of IL-2Rβ -“biased” IL-2 constructs that bind poorly to IL-2Rα in the trimeric receptor of regulatory T cells, and specifically bind to IL-2/15Rβ,γ mimicking the specificity of IL-15 (27, 28) which are reviewed in Klein et al. (29).

**Table 1:** t_1/2_ of IL-15 in different species, different administration^a^.

**Agonist**	**Route**	**t**_****1/2****_**, hr**		
		**Mouse**	**NHP**	**Human**
IL-15	IV	0.64^b^	1.1^c^	2.5^d^
	SC	0.67^e^	2.7^f^	~4^g^
	IP	0.50^h^		
RLI	IP	3^h^		
hetIL-15	IV SC		1.5^j^ 12^j^	
	IP	4^i^		
ALT-803	IV SC	7.5^k^	7.5^l^	0.75- to 5^m^, ^n^ 30^m^^,^ ^n^
NKTR-255	IV	14^o^	30^o^	

a Omissions indicate data is not available.

b Han et al. (18).

c Waldmann et al. (19).

d Conlon et al. (15).

e Zhao et al. (20).

f Sneller et al. (21).

g Miller et al. (22).

h Bessard et al. (23).

i Chertova et al. (9).

j Bergamaschi et al. (24).

k Liu et al. (11).

l Rhode et al. (10).

m Margolin et al. (25).

n Romee et al. (26).

o Kuo (7).

Significantly, and enigmatically, although the half-life extension technologies used often lengthen the t_1/2_ of a protein to a week or longer in humans, the IL-15 agonists studied have systemic elimination t_1/2_s of not much longer than 1 day and usually only several hours (Table 1). Indeed, the only current IL-15 agonists that might be sufficient for QWk injections are the SC administered ALT-803 and the IV-administered PEGylated IL-15, NKTR-255. Interestingly, IV administration of the “long-acting” ALT-803 agonist in humans has a dose-dependent elimination t_1/2_ of ~ 0.75 to 5 h (26), which is not significantly different than the 2.5 h t_1/2_ of IV-administered native IL-15 (15). The dose-dependent elimination rate is likely due to a significant proportion of the drug being consumed by target cells—[i.e., TMDD—as observed with NKTR-255 (13)]. Thus, the longer apparent t_1/2_ of 30 h for SC administered ALT-803 is not due to the retardation of systemic elimination intended by its large size, but rather due to slow absorption from the injection site. Regardless, even with the 30 h t_1/2_ for SC ALT-803 in humans, the level of exposure needed for proliferation and survival of target cells is sustainable for only a few days, and >90% of the drug will be eliminated in 4 days.

Although slow SC absorption provides a simple, practical approach toward achieving half-life extension, it may have unintended shortcomings. For example, SC administration of proteins may or may not have adequate bioavailability. For native IL-15, the SC t_1/2_ in humans is close to that of the IV injection (~4 h for SC vs. 2.5 h for IV), and the bioavailability is estimated from reported data to be near 100% (15, 22). Hence, most of the SC-administered IL-15 is rapidly and completely transferred to the systemic circulation. In contrast, in humans SC ALT-803 shows a long t_1/2_ of ~30 h but a bioavailability of only about 3% (26). Interestingly, the 40 pM C_max_ of the agonist administered SC is some 100-fold lower than that of the same amount injected IV, yet it is significantly more effective at NK and CD8^+^ T cell expansion; clearly, the superior pharmacodynamic effects of SC vs IV administered ALT-803 are due to its longer effective t_1/2_. The very low bioavailability of SC ALT-803 reveals that most (~97%) of the administered drug never reaches the systemic circulation and may activate and be consumed by immune cells near the injection site. Hence, the substantial local toxicity of SC-injected ALT-803—which is not observed with IL-15—may be due to the very high level of the super-agonist deposited at the site of injection and its slow departure. Enigmatically, the problem cannot be solved by simply changing the mode of administration of ALT-803 from SC to IV or intraperitoneal (30) injections since the t_1/2_ of ALT-803 via these routes are so much lower. Whether the other IL-15 super-agonists – such as hetIL-15 and RLI – will face the same conundrum in humans is not known since relevant pharmacokinetic parameters have not yet been reported.

### IL-15 Consumption by a Cytokine Sink

Why do the IL-15 agonists have shorter than expected t_1/2_s? There is abundant evidence for an IL-15-induced “cytokine sink” that increases with proliferation of target immune cells (Figure 1C) and causes commensurate increases in the consumption/clearance of IL-15 (Table 1)—a prototypical example of “drug-induced” or “dynamic” TMDD (14). First, CIV infusion of IL-15 in monkeys and in man initially results in the expected steady state level of serum IL-15 which then decreases ~4- to 5-fold over time (16, 21); this unusual effect can best be explained by a time dependent increase of clearance of IL-15. Second, sequential SC administration of the same doses of the super-agonist hetIL-15 results in decreasing C_max_ and C_min_ upon repeated injections (24); but, when the dose is doubled in each injection by a “doubling step dose” a constant C_max_ and C_min_ can be maintained. This shows that increased hetIL-15 dosing is necessary to satiate the increased appetite for consumption by expanded target cells. Third, pharmacokinetic modeling of cergutuzomab amunaleukin—a CEA targeted immunocytokine containing an IL-15-like IL-2Rβ biased IL-2 mutein—revealed expansion of a drug-induced peripheral sink that increases clearance (27). Fourth, the IL-15/ILRα Fc-fusion XmAb24306 shows low potency but a long t_1/2_, which is in accord with the inverse relationship between cytokine-induced expansion of a peripheral sink and cytokine t_1/2_ (31). Finally, several IL-15 fusions show unexpectedly high rates of systemic elimination that are best explained by a large peripheral sink. The ~114 kDa ALT-803—expected to have a slow systemic elimination rate because of its large size—has an average elimination t_1/2_ of only ~3 h in humans when injected IV; and, connection of RLI to the carboxy terminus of anti-CD20 rituximab—which has a t_1/2_ of about 100 h in mice—results in a large fusion protein with >10-fold lower t_1/2_ than the antibody carrier itself (32). Hence, expansion of target cells by IL-15 agonists is accompanied by a dynamic increased consumption, increased clearance, and decreased lifetime of the agonists.

### Summary

The efficacy of IL-15 agonists in stimulating target immune cell proliferation is paradoxically coupled to its more rapid consumption and clearance. By far, the most efficacious way to deliver IL-15 is by CIV infusion, but this mode of administration is generally impractical. The available IL-15 super-agonists are administered as single injections at intervals of several days to 1 week and are more practical to administer than CIV infusion; however, because of the expanding consumptive sink that accompanies target cell proliferation, they have relatively short systemic half-lives and do not achieve the level of immune cell proliferation and maintenance as CIV infusion. Ideally, what is needed is an IL-15 agonist that is as effective as CIV infusion, but with the convenience and acceptance of single injections at one-week or longer intervals. In a forthcoming report, we will describe our efforts at developing a very slow-releasing hydrogel depot of IL-15 that attempts to achieve this goal.

## Author Contributions

JH wrote and edited the manuscript. DS conceived, wrote, and edited the manuscript. TW provided critical comments, concepts, and insights. All authors read and agreed to the content of this work prior to submission. All authors contributed to the article and approved the submitted version.

## Conflict of Interest

JH and DS are employees and shareholders of ProLynx. The remaining author declares that the research was conducted in the absence of any commercial or financial relationships that could be construed as a potential conflict of interest.
